# Supplementary material for: Comparative genomics provides new insights into the diversity, physiology, and sexuality of the only industrially exploited tremellomycete: Phaffia rhodozyma
Source: BMC Genomics. 2016 Nov 9;17:901. doi: 10.1186/s12864-016-3244-7 (PMC5103461; doi:10.1186/s12864-016-3244-7)
Supplement: Additional file 6: — List of orphan genes with links to PFAM (related to Additional file 1: Table S1). (ZIP 1428 kb) [file 12864_2016_3244_MOESM6_ESM.zip › BLAST_HTML_FTR/G05227_P.html]

BLAST Search Results


```
BLASTP 2.2.27+


Reference:
Stephen F. Altschul, Thomas L. Madden, Alejandro A. Schäffer,
Jinghui Zhang, Zheng Zhang, Webb Miller, and David J. Lipman (1997),
"Gapped BLAST and PSI-BLAST: a new generation of protein database
search programs", Nucleic Acids Res. 25:3389-3402.


Reference for
composition-based statistics:
Alejandro A. Schäffer, L. Aravind, Thomas L. Madden, Sergei
Shavirin, John L. Spouge, Yuri I. Wolf, Eugene V. Koonin, and
Stephen F. Altschul (2001), "Improving the accuracy of PSI-BLAST
protein database searches with composition-based statistics and
other refinements", Nucleic Acids Res. 29:2994-3005.


Database: nr
           71,551,133 sequences; 26,053,659,533 total letters


Query= G05227_P

Length=173
                                                                      Score     E
Sequences producing significant alignments:                          (Bits)  Value

emb|CDZ97944.1|  hypothetical protein [Xanthophyllomyces dendrorh...   328    3e-109
ref|XP_009493343.1|  hypothetical protein H696_01183 [Fonticula a...  38.1    3.8   
ref|XP_011096317.1|  PREDICTED: uncharacterized protein LOC105175...  37.7    4.2   
ref|WP_033879151.1|  conjugal transfer protein [Escherichia coli]...  37.4    5.2   
ref|WP_044537755.1|  conjugal transfer protein [Escherichia coli]     37.0    7.0   
gb|AEE59512.1|  conserved hypothetical protein [Escherichia coli ...  37.0    7.2   


 >emb|CDZ97944.1| hypothetical protein [Xanthophyllomyces dendrorhous]
Length=353

 Score =  328 bits (840),  Expect = 3e-109, Method: Compositional matrix adjust.
 Identities = 163/164 (99%), Positives = 164/164 (100%), Gaps = 0/164 (0%)

Query  9    ERSAFVPLRPSPSVERKPRASPSRTFPSQPQSFLSVQLETSPTSPKIISSSPCTRQALLR  68
            +RSAFVPLRPSPSVERKPRASPSRTFPSQPQSFLSVQLETSPTSPKIISSSPCTRQALLR
Sbjct  190  KRSAFVPLRPSPSVERKPRASPSRTFPSQPQSFLSVQLETSPTSPKIISSSPCTRQALLR  249

Query  69   ISQSDKVSKSSDKSPVKLQYLSTVSERVPADDYTSFVTSSGHHQEAWSAPPGTKSSRKKH  128
            ISQSDKVSKSSDKSPVKLQYLSTVSERVPADDYTSFVTSSGHHQEAWSAPPGTKSSRKKH
Sbjct  250  ISQSDKVSKSSDKSPVKLQYLSTVSERVPADDYTSFVTSSGHHQEAWSAPPGTKSSRKKH  309

Query  129  NFLTDATSSEGRRDFASMPEPVKTMSIAGRKITLWEPPLLSPSS  172
            NFLTDATSSEGRRDFASMPEPVKTMSIAGRKITLWEPPLLSPSS
Sbjct  310  NFLTDATSSEGRRDFASMPEPVKTMSIAGRKITLWEPPLLSPSS  353


>ref|XP_009493343.1| hypothetical protein H696_01183 [Fonticula alba]
 gb|KCV71765.1| hypothetical protein H696_01183 [Fonticula alba]
Length=1189

 Score = 38.1 bits (87),  Expect = 3.8, Method: Compositional matrix adjust.
 Identities = 34/118 (29%), Positives = 56/118 (47%), Gaps = 9/118 (8%)

Query  11   SAFVPLRPSPSVERKPRASPSRTFPSQPQSFLSVQLETSPTSPKIISSSPCTRQALLRIS  70
            S FVPL  + S  R    SP ++      +FL+ ++     + +++++S  TRQALL  +
Sbjct  224  SGFVPLLDT-SARRLVAESPEQSESLAVLAFLAAEI-----TARLLNASDFTRQALLDAA  277

Query  71   QSDKVSKSSDK---SPVKLQYLSTVSERVPADDYTSFVTSSGHHQEAWSAPPGTKSSR  125
             S + S  S +   +P K  YL T  E    D YT  + +        +A PG ++ R
Sbjct  278  ASKRASLQSAEHGPAPSKFSYLLTSYESTVLDPYTYGLFARSLLASGHAAAPGGRAGR  335


>ref|XP_011096317.1| PREDICTED: uncharacterized protein LOC105175546 [Sesamum indicum]
 ref|XP_011096326.1| PREDICTED: uncharacterized protein LOC105175546 [Sesamum indicum]
 ref|XP_011096332.1| PREDICTED: uncharacterized protein LOC105175546 [Sesamum indicum]
 ref|XP_011096341.1| PREDICTED: uncharacterized protein LOC105175546 [Sesamum indicum]
 ref|XP_011096348.1| PREDICTED: uncharacterized protein LOC105175546 [Sesamum indicum]
Length=780

 Score = 37.7 bits (86),  Expect = 4.2, Method: Compositional matrix adjust.
 Identities = 40/150 (27%), Positives = 59/150 (39%), Gaps = 22/150 (15%)

Query  9    ERSAFVPLRPSPSVERKPRASPSRTFPSQPQSFLSVQLETSPTSPKIISSSPCTRQALLR  68
            E SAF+P   S  ++RK  +  +   P  PQ F  + L  +       +  P   + +L+
Sbjct  441  ETSAFLPAVHSKCLDRKTCSRNNE--PKAPQGFTGIDLNIAALEDSAPNGMPMEHEGILQ  498

Query  69   ISQSDKVSKSSDKSPVKLQYLSTVSERVPADDYTS---FVTSSGHHQEAWSAPPGTKSSR  125
             SQS+  SK S    + L  L        AD++T       S  HH E  +    T    
Sbjct  499  DSQSEVDSKRSKNLWIDLNCLYDA-----ADEFTQPSLLPVSENHHVEDLNLNVTTSEGY  553

Query  126  KKHNF---------LTDATSSE---GRRDF  143
            + +N          L + TS     GRRDF
Sbjct  554  RSYNLQRLGKGLQSLGNKTSDSANPGRRDF  583


>ref|WP_033879151.1| conjugal transfer protein [Escherichia coli]
 gb|EZB63252.1| conjugal transfer protein [Escherichia coli O15:H18 str. K1516]
Length=310

 Score = 37.4 bits (85),  Expect = 5.2, Method: Compositional matrix adjust.
 Identities = 29/85 (34%), Positives = 41/85 (48%), Gaps = 10/85 (12%)

Query  78   SSDKSPVKLQYLSTVSERVPADDYT---SFVTSSGHHQEAWSAPPGTKSSRKKHNFLTDA  134
            +  +S V L+  STVS +V  D      +FVT++  H+  W  P GT         +T  
Sbjct  225  AGGRSVVALKLTSTVSRKVVLDPRALQGNFVTATFQHR--WLGPAGTPEDTTTLYLVT--  280

Query  135  TSSEGRRDFASMPEPVKTMSIAGRK  159
               EGR D A + EP +  + AGRK
Sbjct  281  ---EGRPDRAFIAEPARRNATAGRK  302


>ref|WP_044537755.1| conjugal transfer protein [Escherichia coli]
Length=297

 Score = 37.0 bits (84),  Expect = 7.0, Method: Compositional matrix adjust.
 Identities = 26/85 (31%), Positives = 42/85 (49%), Gaps = 10/85 (12%)

Query  78   SSDKSPVKLQYLSTVSERVPADDYT---SFVTSSGHHQEAWSAPPGTKSSRKKHNFLTDA  134
            +  ++ V L+ ++T+S +V  D      +FVT++  H+  W  P GT         +T  
Sbjct  212  AGGRTVVALKLINTISRKVVLDPRALQGNFVTATFQHR--WLGPAGTPEDTTTLYLVT--  267

Query  135  TSSEGRRDFASMPEPVKTMSIAGRK  159
               EGR D A + EP +  + AGRK
Sbjct  268  ---EGRPDRAFIAEPARRNATAGRK  289


>gb|AEE59512.1| conserved hypothetical protein [Escherichia coli UMNK88]
 gb|KDP14817.1| conjugal transfer protein [Escherichia coli]
Length=310

 Score = 37.0 bits (84),  Expect = 7.2, Method: Compositional matrix adjust.
 Identities = 26/85 (31%), Positives = 42/85 (49%), Gaps = 10/85 (12%)

Query  78   SSDKSPVKLQYLSTVSERVPADDYT---SFVTSSGHHQEAWSAPPGTKSSRKKHNFLTDA  134
            +  ++ V L+ ++T+S +V  D      +FVT++  H+  W  P GT         +T  
Sbjct  225  AGGRTVVALKLINTISRKVVLDPRALQGNFVTATFQHR--WLGPAGTPEDTTTLYLVT--  280

Query  135  TSSEGRRDFASMPEPVKTMSIAGRK  159
               EGR D A + EP +  + AGRK
Sbjct  281  ---EGRPDRAFIAEPARRNATAGRK  302


Lambda      K        H        a         alpha
   0.313    0.125    0.359    0.792     4.96 

Gapped
Lambda      K        H        a         alpha    sigma
   0.267   0.0410    0.140     1.90     42.6     43.6 

Effective search space used: 642166500729


  Database: nr
    Posted date:  Sep 23, 2015 12:05 AM
  Number of letters in database: 26,053,659,533
  Number of sequences in database:  71,551,133


Matrix: BLOSUM62
Gap Penalties: Existence: 11, Extension: 1
Neighboring words threshold: 11
Window for multiple hits: 40
```
